# Impact of Proinflammatory Cytokines on the Virulence of Uropathogenic *Escherichia coli*

**DOI:** 10.3389/fmicb.2019.01051

**Published:** 2019-05-09

**Authors:** Ulrik Engelsöy, Ignacio Rangel, Isak Demirel

**Affiliations:** ^1^School of Medical Sciences, Örebro University, Örebro, Sweden; ^2^Nutrition-Gut-Brain Interactions Research Centre, Faculty of Medicine and Health, Örebro University, Örebro, Sweden; ^3^iRiSC - Inflammatory Response and Infection Susceptibility Centre, Faculty of Medicine and Health, Örebro University, Örebro, Sweden

**Keywords:** uropathogenic *Escherichia coli*, proinflammatory cytokines, cross kingdom interaction, virulence, bacterial growth

## Abstract

The effect of a urinary tract infection on the host is a well-studied research field. However, how the host immune response affects uropathogenic *Escherichia coli* (CFT073) virulence is less studied. The aim of the present study was to investigate the impact of proinflammatory cytokine exposure on the virulence of uropathogenic *Escherichia coli.* We found that all tested proinflammatory cytokines (TNF-α, IL-1β, IL-6, IL-8 and IFN-γ) induced an increased CFT073 growth. We also found that biofilm formation and hemolytic activity was reduced in the presence of all proinflammatory cytokines. However, a reduction in siderophore release was only observed in the presence of IL-1β, IL-6 and IL-8. Real time-qPCR showed that all proinflammatory cytokines except TNF-α significantly increased genes associated with the iron acquisition system in CFT073. We also found that the proinflammatory cytokines induced significant changes in type-1 fimbriae, P-fimbriae and gluconeogenetic genes. Furthermore, we also showed, using a *Caenorhabditis elegans* (*C. elegans)* killing assay that all cytokines decreased the survival of *C. elegans* worms significantly. Taken together, our findings show that proinflammatory cytokines have the ability to alter the virulence traits of UPEC.

## Introduction

*Escherichia coli* (*E. coli*) is a ubiquitous member of the intestinal microbiota and it is by far the most common etiological agent in urinary tract infection (UTI). The prototypical pathogenesis for a UTI starts with contamination and colonization of the urethral area with uropathogenic *E. coli* (UPEC). Cystitis is the phase of the infection when bacteria have ascended the urethra and infected the bladder (Flores-Mireles et al., 2015). For a cystitis to arise, UPEC needs to avoid being flushed out by the urine flow. The gene *fimH* codes for the adhesin part of the type-1 fimbriae that mediates binding to mannose motifs prevalent in the urinary tract on the urothelium. The fimH protein also mediates UPEC invasion of the bladder epithelial cells through α3β1 integrin interactions. The type-1 fimbriae are also involved in biofilm formation and establishment of intracellular bacterial colonies (IBC) (Mulvey et al., 1998; Eto et al., 2007; Flores-Mireles et al., 2015). UPEC can also ascend further up the urinary tract and infect the kidneys. P-fimbriae enable adhesion to renal epithelium through attachment to globosides, a type of glycolipid present on cells in the tubuli and collecting ducts (Korhonen et al., 1986; Flores-Mireles et al., 2015). Additional virulence traits important for colonizing the urinary tract are; siderophores (iron scavenger system), α-hemolysin, capsule and biofilm formation (Bower et al., 2005; Subashchandrabose and Mobley, 2015). Another interesting factor in the pathogenesis of UPEC is the bacteria’s metabolic activity. The role that different metabolic pathways play in the pathogenesis and fitness of UPEC during a UTI is not well studied (Subashchandrabose and Mobley, 2015). However, studies have shown that the TCA cycle and gluconeogenesis, but not the glycolysis pathway are important for the fitness of UPEC during UTI (Alteri et al., 2009).

The host response to a UPEC infection is dependent on the innate immune response. Activation of pathogen associated molecular pattern (PAMP)-receptors, primarily TLR4, TLR5, TLR11 (Hagberg et al., 1984; Andersen-Nissen et al., 2007) and NLRP3 (Waldhuber et al., 2016; Demirel et al., 2018) by UPEC will induce an inflammatory response and cytokine secretion. TNF-α, IL-1β, IL-6, IL-8 and IFN-γ are some of the major cytokines being released during UTI (Spencer et al., 2014). Levels of up to 800 (TNF-α), 7000 (IL-1β), 1500 (IL-6), 8000 (IL-8) and 1400 pg/ml (IFN-γ) have been found in the urine of patients with acute cystitis (Sundac et al., 2016). IL-1β is expressed by bladder epithelial cells (Nagamatsu et al., 2015; Demirel et al., 2018) and has been shown to be important for clearance of UPEC (Hertting et al., 2003; Ambite et al., 2016). UPEC isolates can also induce IL-6 from the urothelium (Samuelsson et al., 2004) and a UPEC infection in IL-6 deficient mice showed increased mortality (Khalil et al., 2000). The chemokine IL-8 is essential for the recruitment of neutrophils during a UTI (Godaly et al., 1997, 2000; Hang et al., 2000). Mice lacking IL-8 were unable to clear the infection and developed renal scarring and sepsis (Hang et al., 2000). Both TNF-α and IFN-γ have also been speculated to be important for the clearance of UPEC (Davidoff et al., 1997; Jones-Carson et al., 1999).

The majority of research conducted today in the field of host-pathogen interaction is focused on elucidating how pathogens, with their respective virulence factors, successfully modulate or evade the immune responses to cause infections. However, less is known about how host immune factors like cytokines are affecting the virulence of UPEC by cross kingdom interactions. Mahdavi et al. (2013) showed that *Neisseria meningitidis* can take up TNF-α and IL-8 from the surrounding milieu. These cytokines were shown to be able to bind to certain promotor regions of virulence associated genes in *N. meningitidis* and increase the expression of these genes. This suggest that TNF-α and IL-8 might act as transcription factors, modulating expression of certain genes in *N. meningitidis*. Likewise, Wu et al. (Wu et al., 2005) studied *Pseudomonas aeruginosa* and found that IFN-γ induced increased expression of PA-I, a lectin that is associated with virulence. The increased expression of PA-I was found to be associated with IFN-γ affecting *P. aeruginosa’s* quorum sensing system. Furthermore, it has also been shown that IL-1β, IL-2, IFN-γ and GM-CSF can increase the growth of *E. coli* (Lesouhaitier et al., 2009). IL-1β was shown to specifically bind to virulent strains of *E. coli*, but not avirulent ones, and increase their growth. This increased growth was inhibited by IL-1RA and by heat inactivating IL-1β (Porat et al., 1991). However, with exception for the effects on bacterial growth, little is known about how proinflammatory cytokines affect the expression of UPEC virulence factors. The aim of the present study was to investigate the impact of proinflammatory cytokine exposure on the virulence of UPEC.

## Materials and Methods

### UPEC Strain and Bacterial Growth Assay

CFT073 is a whole genome sequenced uropathogenic *E. coli* strain isolated from a patient with pyelonephritis (Mobley et al., 1990). CFT073 was maintained on tryptic soy agar and grown in Lysogeny broth (Difco Laboratories, Detroit, MI, United States) overnight on shake (150 rpm) at in 37°C prior to experiments. CFT073 (1^∗^10^6^ CFU/mL) was then grown in minimal salt medium (MSM, 1.3% [wt/vol] Na_2_HPO_4_, 0.3% KH_2_PO_4_, 0.05% NaCl, and 0.1% NH_4_Cl supplemented with 20 mM glucose, 2 mM MgSO_4_, 100 μM CaCl_2_, and 0.25% Casamino Acids) with or without the presence of TNF-α (T6674), IL-1β (I9401), IL-6 (I1395), IL-8 (I1645) or IFN-γ **(**SRP3058) (0.5, 1, 10, 20 or 40 ng/ml, all from Sigma-Aldrich, St. Louis, MO, United States) in a 96-well plate. All cytokines were with no additives. The microplate was incubated at 37°C for 24 h and the growth was measured by optical density at 600 nm every 10 min using a spectrophotometer (Cytation 3, Biotek Inc, Winooski, VT, United States).

### Human Bladder Epithelial Cells

The human bladder epithelial cell line 5637 was acquired from the American Type Culture Collection (Manassas, VA, United States). Cells were grown in Dulbecco’s Modified Eagle Medium (DMEM) (Lonza, Basel, Switzerland) supplemented with 2 mM L-glutamine, 10% fetal bovine serum (FBS) and 1 mM non-essential amino acids (all from Thermo Fisher Scientific, Waltham, MA, United States) at 37°C in a 5% CO_2_ atmosphere. The cell medium was replaced with DMEM containing 2% FBS, 1 mM non-essential amino acids and 2 mM L-glutamine during experiments.

### Biofilm Assay

After measuring bacterial growth for 24 h, the same plate was used to evaluate biofilm formation. The wells were washed with sterile RO-water 3 times after the 24-h incubation. The plate was thereafter left to dry for 45 min in room temperature and the biofilm was stained with 0.1% crystal violet for 20 min. The plate was left to dry overnight after 4 additional washes with RO-water. The crystal violet was then destained with 95% ethanol and the absorbance was quantified at 540 nm by spectrophotometry (Sahlberg Bang et al., 2016) (Multiskan Ascent, Thermo Labsystems, Helsinki, Finland). The data is presented as % of unstimulated CFT073. The dotted line in the figure illustrates the unstimulated control.

### Hemolysis Assay

Whole blood was collected in heparinized tubes and the erythrocytes were washed 2 times with PBS and diluted to a final concentration of 0.8% (v/v) in MSM. This study was carried out in accordance with the recommendations of both the Declaration of Helsinki and the Swedish national board of health and welfare. All subjects gave written informed consent in accordance with the Declaration of Helsinki. The protocol was approved by the regional ethics review board in Uppsala, Sweden (Dnr 2015/437). The erythrocytes (0.8%) were then stimulated with CFT073 (5^∗^10^6^ CFU/mL) in the presence or absence of TNF-α, IL-1β, IL-6, IL-8 or IFN-γ (0.5 or 40 ng/ml) in a 24-well plate. The microplate was incubated at 37°C on a shaker (100 rpm) for 6 h and the hemoglobin leakage was measured by absorbance at 404 nm (Multiskan Ascent, Thermo Labsystems) (Shin et al., 1999). Triton X-100 (Sigma-Aldrich) lysed erythrocytes were used as a positive control. The data is presented as % of unstimulated CFT073. The dotted line in the figure illustrates the unstimulated control.

### Siderophore Assay

CFT073 (1^∗^10^6^ CFU/mL) was grown in MSM with or without the presence of TNF-α, IL-1β, IL-6, IL-8 or IFN-γ (0.5 or 40 ng/ml) in a 96-well plate at 37°C for 6 h. The amount of released bacterial siderophores was evaluated by SideroTec Assay TM (Emergen Bio, Ireland) according to manufacturer instructions. The data is presented as % of unstimulated CFT073. The dotted line in the figure illustrates the unstimulated control.

### Adhesion Assay

CFT073 (1^∗^10^6^ CFU/mL) with a eGFP expressing plasmid was grown in MSM with or without the presence of TNF-α, IL-1β, IL-6, IL-8 or IFN-γ (0.5 ng/ml or 40 ng/ml) in a 96-well plate at 37°C for 6 h. After 6 h, the bacterial amount was adjusted and used to infect the bladder epithelial cell line 5637 for 3 h at 37°C with 5% CO_2_. The wells were then washed with PBS and the adhered eGFP expressing CFT073 were quantified and imaged with a Cytation 3 plate reader (BioTek, Winooski, VT, United States) (Demirel et al., 2018). Adhesion is presented as mean fluorescence intensity (MFI).

### RNA Isolation, cDNA Synthesis and Real Time-qPCR

CFT073 (1^∗^10^6^ CFU/mL) was grown in MSM with or without the presence of TNF-α, IL-1β, IL-6, IL-8 or IFN-γ (0.5 or 40 ng/ml) in a 96-well plate for 6 h in 37°C. The bacteria were treated with RNAlater (Sigma-Aldrich) prior to isolation of total RNA using the E.Z.N.A^®^ Total RNA Kit I (Omega Bio-tek, Inc., Norcross, GA, United States) according to manufacturer instructions. DNA contamination was removed by DNase digestion (TURBO DNase, Life technologies, MA, United States) according to manufacturer instructions. 100 ng of total RNA was reverse transcribed to cDNA using the High Capacity cDNA Reverse Transcription Kit for single-stranded cDNA synthesis (Applied Biosystems, CA, United States). Maxima SYBR Green qPCR Master Mix (Thermo Fisher Scientific, MA, United States) was used for the real time-qPCR. 5 ng of templet cDNA and 250 nM of primer (Table 1, Eurofins MWG Synthesis GmbH, Ebersberg, Munich, Germany) was used in the real time-qPCR. A CFX96 Touch^TM^ Real-Time PCR Detection System (Biorad, CA, United States) was used for the amplification using the following protocol: initial denaturation at 95°C for 10 min, 40 cycles of denaturation at 95°C for 15 s followed by annealing at 60°C for 30 s and extension at 72°C for 30 s. The qPCR was followed by a dissociation curve analysis between 60 and 95°C. The *C*_t_ values were analyzed by the ΔΔ*C*_t_ method and normalized to the endogenous control *gapA* (glyceraldehyde 3-phosphate dehydrogenase A). Fold difference was calculated as 2^-ΔΔC_t_^.

**Table 1:** Primers used for real-time qPCR.

Gene symbol	Oligonucleotide sequences (5′-3′)
*iutA*	*F:* AAAGAGCTGAAAGACGCACTGG *R:* TGTCGGAACGTGAAGAGTTGAG
*fimH*	*F:* GTGCCAATTCCTCTTACCGTT *R:* TGGAATAATCGTACCGTTGCG
*iroN*	*F:* ATTACCAAACGTCCCACCAACG *R:* AAACGCGTGGTAAGAGCATCAC
*iha*	*F:* TGCGAATAACCACTCTGGCTTC *R:* TAATCACAGAAACACTGGCGGC
*chuA*	*F:* AAGGCGTTGCCCAATACCAGAGTA *R:* TATTCCGATCGCTCACAGTGGCTT
*papC*	*F:* GTGGCAGTATGAGTAATGACCGTTA *R:* ATATCCTTTCTGCAGGGATGCAATA
*pgi*	*F:* CTCTGGCGAGAAGATCAACC
	*R:* TCACCGGAAATAATCGCTTC
*ppsA*	*F:* GCAAAACAGGCCGTACAAAT
	*R:* CAGCGTATAACGCTCCATGA
*frdA*	*F:* CAACACCGACCTGCTCTACA
	*R:* GCGGCAGCGTAGTAATCTTC
*hlyA*	*F:* TCACGAATTTCCTCACCGGGAGTT *R:* TTATGAAGAGGGAAAGCGGCTGGA
*flu*	*F:* TAACAGCGTCCGTCTCAGCATTCA *R:* AACATCAACGGAAGAATGGCCTGC
*gapA*	*F:* AAGTTGGTGTTGACGTTG *R:* AGCGCCTTTAACGAACATCG


### *C. elegans* Killing Assay

The Bristol wild type N2 strain of *C. elegans* (Caenorhabditis Genetics Center, University of Minnesota, United States) was maintained on nematode growth medium plates seeded with a lawn of *E. coli* OP50 at 21°C. Prior to experiments, *C. elegans* were alkaline/hypochlorite-synchronized (0.25M NaOH, 1% HOCl) and maintained on nematode growth medium plates for 48 h at 21°C to reach L4 stage. CFT073 (1^∗^10^6^ CFU/mL) was grown in MSM with or without the presence of TNF-α, IL-1β, IL-6, IL-8 or IFN-γ (0.5 ng/ml) in a 96-well plate at 37°C for 6 h. The bacteria were then cooled down to 21°C prior to *C. elegans* addition. Age synchronized L4 worms were washed with M9 buffer and 10 worms were then transferred to respective well of bacteria that had been pre-grown for 6 h. After the addition of the worm to the wells, *C. elegans* and the bacteria were incubated together for 1 h at 21°C. A worm was considered dead when it failed to respond to touch. Dead worms were also visualized with 1 μM Sytox Green (ThermoFisher Scientific) using a spectrophotometer (Cytation 3) (Gill et al., 2003). The data is presented as survival % of unstimulated CFT073. The dotted line in the figure illustrates the unstimulated control.

### Statistical Analyses

The differences between groups were analyzed with one-way ANOVA followed by Bonferroni test. Differences were considered statistically significant when *p* < 0.05 vs. unstimulated bacteria. Data are presented as mean ± standard error of the mean (SEM), n = number of independent biological experiments. Equality of group variances was tested for all the different variables and no significant differences were found according to the Brown-Forsythe test in spite of the limited n number. Therefore, we decided to analyse the data applying ANOVA tests.

## Results

### Cytokines Induce Increased UPEC Growth

**FIGURE 1:**
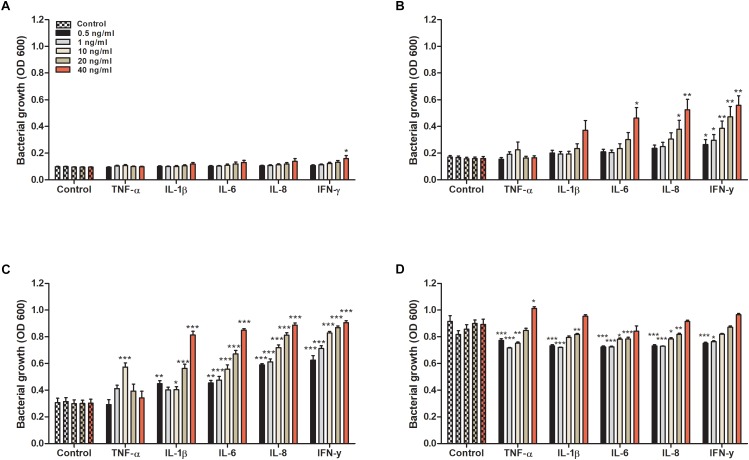
Bacterial growth with or without the presence of TNF-α, IL-1β, IL-6, IL-8 or IFN-γ (0.5, 1, 10, 20, or 40 ng/ml) for 4 **(A)**, 6 **(B)**, 8 **(C)**, and 24 h **(D)**. Control represents unstimulated bacteria. Data are presented as mean ± SEM of *n* = 4 independent experiment. Statistical significance is denoted with asterisks: ^∗^*p* < 0.05, ^∗∗^*p* < 0.01, and ^∗∗∗^*p* < 0.001 vs. unstimulated bacteria.

We started by evaluating the effect that cytokines had on the growth of CFT073. We found that all evaluated cytokines had an overall effect on the growth of CFT073. Growth data collected at 4, 6, 8, and 24 h are presented in Figure 1. No statistical difference was observed except for IFN-γ after 4 h (Figure 1A). Nevertheless, IL-1β and above all IL-6, IL-8 and IFN-γ increased the growth of CFT073 in a concentration dependent manner in the range 4 to 8 h of stimulation compared to the unstimulated controls (Figure 1A–C). Exposure to TNF-α increased the growth significantly only at 10 ng/ml after 8 h of stimulation. Interestingly, 20 and 40 ng/ml of TNF-α did not induce increased growth after 8 h (Figure 1C). After 24 h of stimulation, the stationary phase was reached. At this phase, the cytokine stimulated bacteria showed similar or slightly reduced growth compared to the controls with all cytokines except with TNF-α at 40 ng/ml where the growth was increased (Figure 1D). In addition, we found that a combination treatment of TNF-α, IL-1β, IL-6, IL-8 and IFN-γ at 0.5 ng/ml, induced a higher growth than the respective cytokines alone after 6 and 8 h (Supplementary Figure S1).

### Reduced Biofilm Formation, Hemolytic Activity and Siderophore Release

**FIGURE 2:**
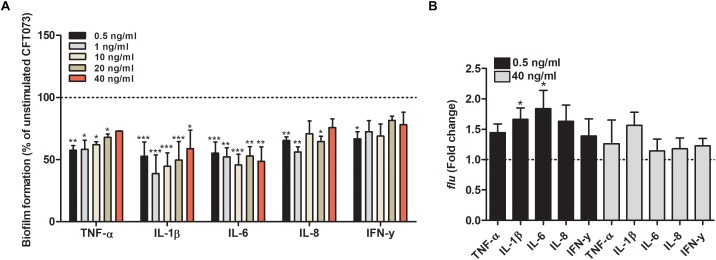
Bacterial biofilm formation **(A)** and real time-qPCR analysis of *flu* **(B)** mRNA expression in the presence or absence of TNF-α, IL-1β, IL-6, IL-8 or IFN-γ (A: 0.5, 1, 10, 20 or 40 ng/ml, B: 0.5 or 40 ng/ml) after 6 h **(B)** or 24 h **(A)**. Dotted line represents data from unstimulated bacteria. Data are presented as mean ± SEM of *n* = 3 independent experiment. Statistical significance is denoted with asterisks: ^∗^*p* < 0.05, ^∗∗^*p* < 0.01 and ^∗∗∗^*p* < 0.001 vs. unstimulated bacteria.

**FIGURE 3:**
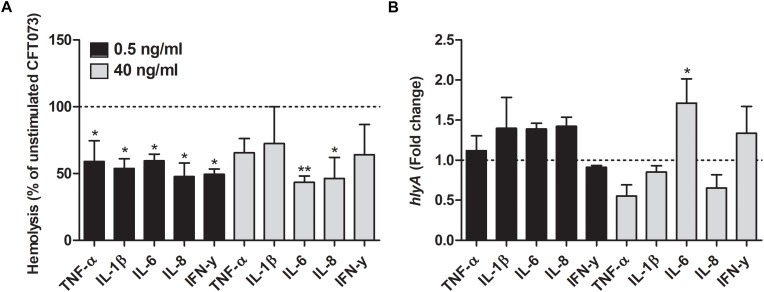
Hemolytic activity **(A)** and real time-qPCR analysis of *hlyA* **(B)** mRNA expression in the presence or absence of TNF-α, IL-1β, IL-6, IL-8 or IFN-γ (0.5 or 40 ng/ml) after 6 h. Dotted line represents data from unstimulated bacteria. Data are presented as mean ± SEM of *n* = 3 independent experiment. Statistical significance is denoted with asterisks: ^∗^*p* < 0.05 and ^∗∗^*p* < 0.01 vs. unstimulated bacteria.

**FIGURE 4:**
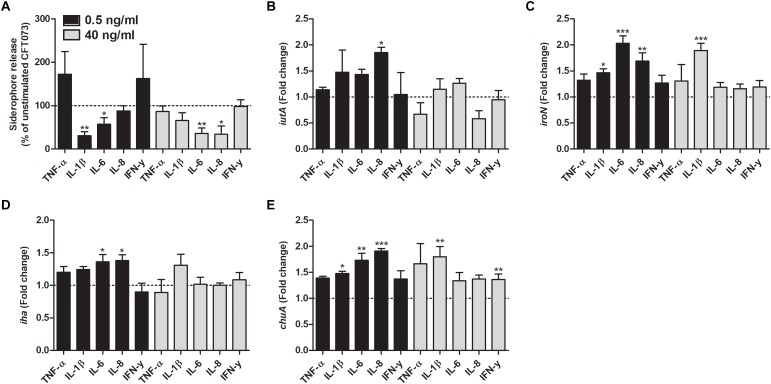
Siderophore release **(A)** and real time-qPCR analysis of *iutA* **(B)**, *iroN* **(C)**, *iha* **(D)** and *chuA* **(E)** mRNA expression in bacteria in the presence or absence of TNF-α, IL-1β, IL-6, IL-8 or IFN-γ (0.5 or 40 ng/ml) after 6 h. Dotted line represents data from unstimulated bacteria. Data are presented as mean ± SEM of *n* = 3–4 independent experiment. Statistical significance is denoted with asterisks: ^∗^*p* < 0.05, ^∗∗^*p* < 0.01 and ^∗∗∗^*p* < 0.001 vs. unstimulated bacteria.

We proceeded with evaluating the effects that cytokines had on biofilm formation, hemolytic activity and siderophore release from CFT073. Biofilm formation was found to be significantly reduced by all cytokines at least on one concentration compared to unstimulated controls (Figure 2A). IL-1β and IL-6 induced some of the largest effects with a reduction in biofilm formation in some concentrations of about 50% compared to unstimulated control. Interestingly, only 0.5 ng/ml of IFN-γ induced a significantly reduced biofilm formation compared to controls (Figure 2A). However, we found that the biofilm associated gene *flu* was significantly increased in the presence of IL-1β and IL-6 compared to unstimulated control (Figure 2B). In addition, we also found that all the cytokines at 0.5 ng/ml decreased the CFT073 hemolytic activity compared to unstimulated controls after 6 h. We found that only IL-6 and IL-8 significantly decreased hemolysis at 40 ng/ml (Figure 3A). However, no significant downregulation of *hlyA* gene expression was observed in the presence of the proinflammatory cytokines. We only found that IL-6 at 40 ng/ml could significantly increase the expression of *hlyA* compared to unstimulated control. (Figure 3B). We also found that IL-1β and IL-6 at 0.5 ng/ml and IL-6 and IL-8 at 40 ng/ml decreased siderophore release from CFT073 compared to unstimulated controls (Figure 4A).

### Cytokines Alter the Gene Expression of Iron Acquisition Systems in CFT073

We investigated the effect that cytokines had on the gene expression of iron acquisition systems. We found that IL-8 at a concentration of 0.5 ng/ml induced a significantly increased expression in all the genes analyzed compared to unstimulated control after 6 h (Figure 4B–D). Furthermore, iroN transcription was found to be significantly increased by IL-1β and IL-6 at 0.5 ng/ml and by IL-1β at 40 ng/ml compared to unstimulated control (Figure 4C). We also found that the iha transcription was significantly elevated by IL-6 at 0.5 ng/ml compared to unstimulated control (Figure 4D). Finally, we also showed that chuA gene expression was increased by IL-1β and IL-6 at 0.5 ng/ml and IL-1β and IFN-γ at 40 ng/ml compared to control (Figure 4E).

### Altered Fimbriae and Metabolic Gene Expression by Cytokines

**FIGURE 5:**
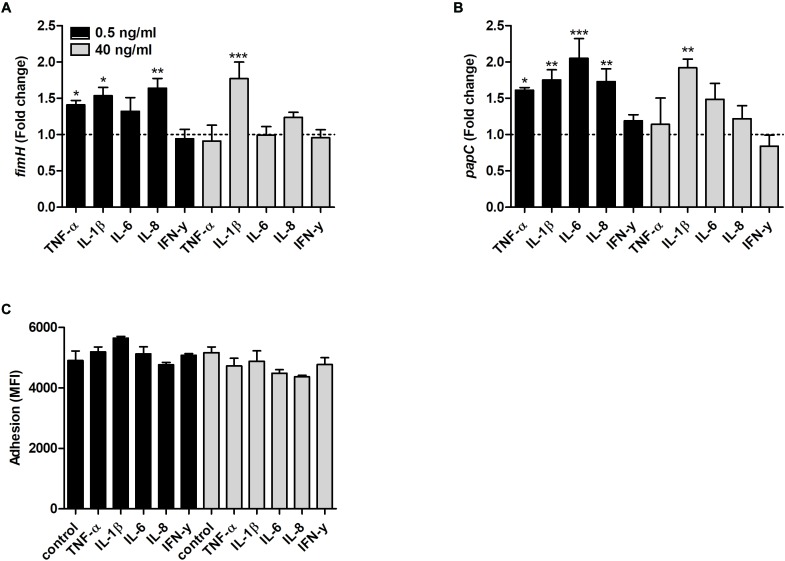
Real time-qPCR analysis of *fimH* **(A)** and *papC* **(B)** mRNA expression in bacteria in the presence or absence of TNF-α, IL-1β, IL-6, IL-8 or IFN-γ (0.5 or 40 ng/ml) after 6 h. Dotted line represents data from unstimulated bacteria. Data are presented as mean ± SEM of *n* = 4 independent experiment. CFT073 (eGFP) adhesion of bladder epithelial cells in the presence or absence of TNF-α, IL-1β, IL-6, IL-8 or IFN-γ (0.5 or 40 ng/ml) after 3 h. **(C)** Adhesion is presented as mean fluorescence intensity (MFI). Statistical significance is denoted with asterisks: ^∗^*p* < 0.05, ^∗∗^*p* < 0.01 and ^∗∗∗^*p* < 0.001 vs. unstimulated bacteria.

We found that the *fimH* transcription was significantly increased by TNF-α, IL-1β and IL-8 at 0.5 ng/ml and by IL-1β at 40 ng/ml compared to unstimulated control after 6 h (Figure 5A). In addition, the *papC* gene expression was also increased by TNF-α, IL-1β, IL-6 and IL-8 at 0.5 ng/ml and by IL-1β at 40 ng/ml compared to unstimulated control (Figure 5B). However, we could not find any significant increased adhesion of CFT073 to the bladder epithelial cells line 5637 in the presence of the proinflammatory cytokines compared to control cells (Figure 5C).

**FIGURE 6:**
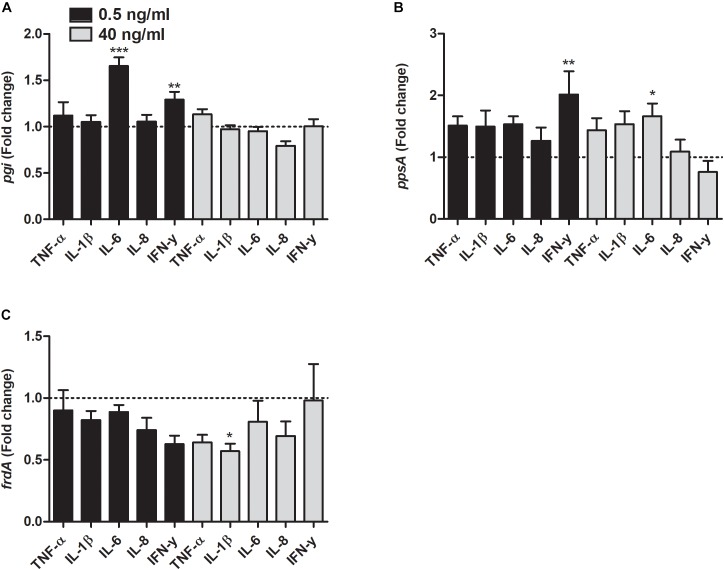
Real time-qPCR analysis of *pgi* **(A)**, *ppsA* **(B)**, and *frdA* **(C)** mRNA expression in bacteria in the presence or absence of TNF-α, IL-1β, IL-6, IL-8, or IFN-γ (0.5 or 40 ng/ml) after 6 h. Dotted line represents data from unstimulated bacteria. Data are presented as mean ± SEM of *n* = 4 independent experiment. Statistical significance is denoted with asterisks: ^∗^*p* < 0.05, ^∗∗^*p* < 0.01 and ^∗∗∗^*p* < 0.001 vs. unstimulated bacteria.

As for the metabolic genes, the transcription of *pgi* was significantly increased by IL-6 and IFN-γ at 0.5 ng/ml compared to unstimulated control (Figure 6A). In addition, the transcription of *ppsA* was also increased by IFN-γ at 0.5 ng/ml and IL-6 at 40 ng/ml compared to unstimulated control (Figure 6B). Finally, we found that the *frdA* transcription was significantly decreased by IL-1β at 40 ng/ml compared to unstimulated control (Figure 6C).

### Increased CFT073 Virulence in the Presence of Cytokine

**FIGURE 7:**
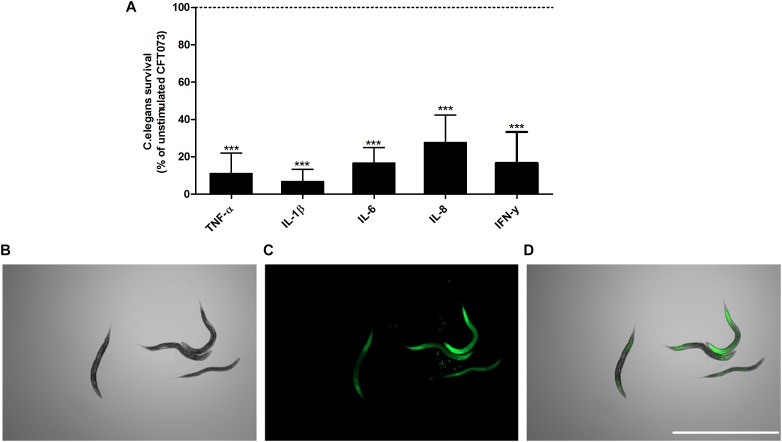
CFT073 mediated *C. elegans* killing in the presence or absence of TNF-α, IL-1β, IL-6, IL-8 or IFN-γ (0.5 ng/ml) **(A)**. Dotted line represents data from unstimulated bacteria. Data are presented as mean ± SEM of *n* = 3 independent experiment. Statistical significance is denoted with asterisks: ^∗^*p* < 0.05, ^∗∗^*p* < 0.01 and ^∗∗∗^*p* < 0.001 vs. unstimulated bacteria. Immunofluorescence staining of dead worm with 1 μM Sytox Green **(B–D)**. Scale bar: 1000 μm.

Next, we proceeded with evaluating the virulence of CFT073 in the presence of cytokines with an *in vivo* *C. elegans* infection model. This was done to assess the combined significance our findings have on the total virulence of CFT073. We found that CFT073 in the presence of all cytokines at 0.5 ng/ml significantly decreased the survival of *C. elegans* worms compared to unstimulated controls (Figure 7A). The dead worms were also visualized by sytox green uptake (Figure 7B–D). The cytokines *per se* did not induce any *C. elegans* toxicity (data not shown).

## Discussion

Understanding the interaction between UPEC and our immune system is crucial as treatment options are heavily compromised due to antibiotic resistance. In this study we focused on investigating the cross kingdom effects of proinflammatory cytokines on the virulence of UPEC. We chose to focus on proinflammatory cytokines due to their primary induction by UPEC and because they have been linked to the progression of the infection (Davidoff et al., 1997; Godaly et al., 1997; Jones-Carson et al., 1999; Khalil et al., 2000; Hertting et al., 2003; Spencer et al., 2014; Ambite et al., 2016; Sundac et al., 2016). The concentration range investigated was 0.5–40 ng/ml, as these cytokines are found in the urine in a concentration range of 0.5–10 ng/ml, however, urine is very diluted (Sundac et al., 2016). The concentration that an adhered bacteria (to epithelial cells) can be exposed to can be much higher in the micro-milieu, hence the choice of higher concentrations also.

Our findings showed a general increase in UPEC growth, concentration dependent for IL-1β, IL-6, IL-8 and IFN-γ, but not TNF-α. In addition, a combination of all cytokines increased UPECs growth more than the respective cytokines alone. It has previously been shown that IL-1β, TNF-α, GM-CSF, IL-2 and IFN-γ increase the growth of *E. coli* (Porat et al., 1991; Lesouhaitier et al., 2009), suggesting that *E. coli* can sense cytokines and alter behavior. As of now, we know that IL-1β (Porat et al., 1991) and TNF-α (Luo et al., 1993) can bind to specific receptors on the surface of *E. coli* and alter the bacterial behavior. However, further investigation is needed to elucidate the mechanisms behind the effects of IL-6, IL-8, and IFN-γ. Interestingly, in our study TNF-α only induced a significantly increased growth at 10 ng/ml, but not at lower or higher concentrations, which might suggest a more complex mechanism. The cross kingdom effect of cytokines is not only limited to *E. coli*. Several studies have found that cytokines can affect the growth and virulence of e.g., *Shigella flexneri* (Luo et al., 1993)*, Pseudomonas* (Wu et al., 2005)*, N. meningitidis* (Mahdavi et al., 2013) and *Yersinia pestis* (Zavyalov et al., 1995). Our results indicate that proinflammatory cytokines induce an increased growth of the UPEC strain CFT073, which may promote persistence in the urinary tract.

We also found that biofilm formation was significantly reduced by all tested cytokines. This is in contrast to the initial planktonic growth increase that was observed. This suggests that in the presence of proinflammatory cytokines, UPEC favors planktonic growth rather than biofilm formation. The expression of the biofilm associated genes *fimH* and *flu* (Wang et al., 2016) cannot explain the reduced biofilm formation, as these genes were upregulated in the presence of the cytokines. Biofilm formation during a UTI infection is associated with protection of UPEC from environmental conditions, antimicrobial agents and the host immune response (Mah and O’Toole, 2001; Hatt and Rather, 2008). However, the increased planktonic growth may favor an increased invasion of bladder epithelial cells. Increased number of planktonic bacteria has been associated with increased invasion and formation of intracellular bacterial communities in bladder epithelial cells (Hannan et al., 2012). The presence of intracellular bacterial communities in the bladder is associated with UPEC persistence, decreased bacterial clearance and recurrent UTI (Justice et al., 2006; Rosen et al., 2007; Blango and Mulvey, 2010).

We proceeded with evaluating the effects that proinflammatory cytokines have on CFT073 hemolytic activity. We found that all cytokines decreased the hemolytic activity of CFT073, but not the gene expression of *hlyA*. Hemolysin in UPEC is encoded by the hlyCABD operon and produced in a pro-toxin form that needs to be modified by the acyltransferase HlyC for cytotoxic activity. Exporting the toxin is then mediated by the inner membrane components HlyB and HlyD along with the outer membrane protein TolC (Justice and Hunstad, 2012). In order to understand the underlining mechanism of our novel findings, further investigations are needed to elucidate the effects cytokines have on the whole hlyCABD operon. The hemolytic activity of UPEC is important to evade the host immune response and for colonization of the urinary tract. α-hemolysin has a dual concentration-dependent effect on bladder epithelial cells. At a high concentration, α-hemolysin lysis host cells to acquire nutrients and allow bacteria to cross mucosal barriers. The host reacts by an induction of IL-1β. However, a low concentration α-hemolysin suppresses NF-κB activation and IL-6 release from bladder epithelial cells (Keane et al., 1987; Ristow and Welch, 2016; Demirel et al., 2018). This suggests that proinflammatory cytokines may reduce the lytic activity of α-hemolysin in favour of the immunomodulatory effects.

Iron is considered to be essential for UPEC survival and pathogenicity in the urinary tract (Subashchandrabose and Mobley, 2015). However, the low levels of soluble iron in the urinary tract are considered a growth limiting factor (Roos et al., 2006). The acquisition of iron by UPEC is mediated by several mechanisms such as ferrous iron transporters, outer membrane receptors for heme and through ferric iron chelators called siderophores (Cho et al., 2008). Our results indicate that cytokines have a different effect on these mechanisms. We observed that IL-1β, IL-6 and IL-8 decreased siderophore release from CFT073. However, we also found that IL-8 increased the expression of the aerobactin uptake receptor IutA. IL-1β, IL-6 and IL-8 increased the expression of the salmochelin uptake receptor IroN. IL-6 and IL-8 increased the expression of the enterobactin uptake receptor Iha. IL-1β, IL-6, IL-8, and IFN-γ increased the expression of the heme uptake receptor ChuA (Yep et al., 2014). It is interesting that IL-1β, IL-6, and IL-8 decrease siderophore release and simultaneously increase the expression of uptake receptors. A potential explanation to this observation is that, if these cytokines, as a host response, reduce the release of siderophores, UPEC counteracts by upregulating uptake receptors. The observed growth increase induced by these cytokines supports this hypothesis. Taken together, these results show that proinflammatory cytokines alter the iron acquisition systems in UPEC, which may have an effect on UPEC pathogenicity.

UPECs ability to adhere and invade epithelial cells is crucial for the colonization of the urinary tract. It is known that UPEC utilizes P-fimbriae for colonization of the kidney and type-1 fimbriae for colonization of the bladder (Korhonen et al., 1986; Flores-Mireles et al., 2015). We found that the type-1 fimbriae (*fimH*) and the P-fimbriae (*papC*) were upregulated by all cytokines except for IFN*-*γ. However, we did not observe a significant increased CFT073 adhesion to bladder epithelial cells after 3 h. This might be associated with the time delay between gene expression and fimbriae synthesis. It has been shown that the type-1 fimbriae expression in planktonic UPEC *in vivo* is downregulated to save energy for growth. However, type-1 fimbriae were shown to be highly expressed on the sessile UPEC population attached to bladder epithelial cells (Staerk et al., 2016). Hence, UPECs ability to sense proinflammatory cytokines and upregulate *fimH* and *papC*, in combination with the increased planktonic growth may indicate a promoted persistence and colonization of the urinary tract.

We proceeded with investigating the effects proinflammatory cytokines have on the metabolic pathways of UPEC. We found that *ppsA* (encoding for phosphoenolpyruvate synthase, a unique enzyme involved in gluconeogenesis) was upregulated by IFN-γ and IL-6, although at different concentrations. One explanation for this observation may be that some proinflammatory cytokines can be used as growth substrate by UPEC. This could be a contributing factor to the increased UPEC growth that we observed. This is supported by previous findings showing that UPEC in urine consumes primarily amino acids and peptides as carbon sources instead of glucose (Alteri et al., 2009). On the other hand, we did not see a pattern of genetic upregulation of *ppsA* as the cytokine concentrations increased. Rather, an inverse trend was observed in the case of IFN-γ. Therefore, other processes such as colony-forming ability, which has been associated to ppsA (Tamura et al., 2016) may instead be linked to the gene regulation of this enzyme as a result of UPEC exposure to some proinflammatory cytokines. Furthermore, we also found that the gene expression of *pgi* (encoding for glucose-6-phosphate isomerase), a glycolytic enzyme, was only increased by IL-6 and IFN-γ. It has previously been shown that the TCA cycle and gluconeogenesis, but not glycolysis, are important for the fitness of UPEC in mice (Alteri et al., 2009; Subashchandrabose and Mobley, 2015). However, the role that the glycolytic pathway plays in human UTI is not well studied. Hence, further studies are needed to clarify what effect the increased expression of *pgi* induced by IL-6 and IFN-γ has on UPEC virulence. We also found a reduction in the expression of *frdA*, the gene encoding for the catalytic subunit of fumarate reductase, an enzyme used for anaerobic respiration. This suggests that in the presence of proinflammatory cytokines aerobic respiration was preferred. That was not surprising given that oxygen access was adequate during our experiments. Furthermore, it has previously been shown that fumarate reductase is not important for the fitness of UPEC in the urinary tract (Alteri et al., 2015). Taken together, these data show that proinflammatory cytokines have the ability to alter the expression of different metabolic pathways in UPEC. Knowledge of these alterations may in the future help us limit the ability of UPEC to acquire alternative carbon sources.

In order to comprehend how the respective virulence alteration induced by proinflammatory cytokines contributes to the total virulence of CFT073, an *in vivo C. elegans* infection model was used. *C. elegans* has previously been used to evaluate the virulence of UPEC. The study showed that there is a significant correlation between the virulence of UPEC in *C. elegans* and in a murine model (Diard et al., 2007). We found that all the cytokines at 0.5 ng/ml decreased the survival of *C. elegans* after 6 h of growth. Hence at 6 h, no difference in bacterial growth could explain these observation. In addition, it is interesting that α-hemolysins lytic activity was decreased after 6 h, but the toxicity against *C. elegans* was increased. It could be speculated that the increased expression of fimbriae can contribute to the increased virulence of CFT073. However, there are several virulence factors expressed by CFT073 that we have not investigated. Two of these are the vacuolating autotransporter toxin (Vat) and secreted autotransporter toxin (Sat). Both of these toxins have been shown to induce tissue damage (Wiles et al., 2008). In all, we have shown that all proinflammatory cytokines tested in this study, increased the total virulence of CFT073.

Understanding the underlining mechanisms by which proinflammatory cytokines alter the virulence of UPEC is very important. In UPEC, there are some known global regulators of gene expression (that have been reviewed by professor Mobley) that could be potential cytokine targets. One could speculate that the PhoPQ two-component regulatory system, associated with regulating 11% of CFT073 genome could be a potential cytokine target. In addition, the Histone-Like Nucleoid Structuring Protein (H-NS), a DNA-binding protein acting as a global transcriptional regulator in UPEC, is known to regulate genes involved in motility, α-hemolysin production, fimbriae expression and iron uptake. Furthermore, the novel quorum-sensing (Qse) system has been proposed as an interkingdom-signaling system in *E. coli*. This system is also found in UPEC. QseB is a transcriptional regulator that affects the expression of target genes including curli and flagella (Subashchandrabose and Mobley, 2015). All these systems are interesting to evaluate as cytokine targets in future studies.

There is today an urgent need for the development of alternative treatments suitable for targeting bacteria that are resistant to traditional antibiotics. One of the proposed strategies is to inhibit the virulence of UPEC and hence reduce the selection pressure on the bacteria (Brannon and Hadjifrangiskou, 2016). Understanding how host immune factors like proinflammatory cytokines affects the virulence of UPEC by cross kingdom interactions is important for the development of alternative treatments strategies. If our immune system is triggering UPEC to become more virulent, as our results suggests, limiting these immune responses may protect the urinary tract from colonization. Although we have found that proinflammatory cytokines have the ability to change the virulence of UPEC, further studies are needed in order to understand the mechanism behind these findings.

## Ethics Statement

This study was carried out in accordance with the recommendations of both the Declaration of Helsinki and the Swedish national board of health and welfare. All subjects gave written informed consent in accordance with the Declaration of Helsinki. The protocol was approved by the regional ethics review board in Uppsala, Sweden (Dnr 2015/437).

## Author Contributions

IR and ID design the study. All authors conducted the experiments, analyzed the data, drafted the manuscript, and read and approved the final manuscript.

## Conflict of Interest Statement

The authors declare that the research was conducted in the absence of any commercial or financial relationships that could be construed as a potential conflict of interest.
