# Supplementary material for: Impact of Proinflammatory Cytokines on the Virulence of Uropathogenic Escherichia coli
Source: Front Microbiol. 2019 May 9;10:1051. doi: 10.3389/fmicb.2019.01051 (PMC6520761; doi:10.3389/fmicb.2019.01051)
Supplement: Supplementary file 1 [file Data_Sheet_1.docx]

**Figure S1:** Bacterial growth with or without the presence of TNF-α, IL-1β, IL-6, IL-8 or IFN-γ (0.5 ng/ml) for 4, 6, 8 and 24 h. Control represents unstimulated bacteria. Data are presented as mean ± SEM of n=3 independent experiment. Statistical significance is denoted with asterisks: *=p<0.05, **=p<0.01 and ***=p<0.001.
